# Potential Probiotic Acceptability of a Novel Strain of *Paenibacillus konkukensis* SK 3146 and Its Dietary Effects on Growth Performance, Intestinal Microbiota, and Meat Quality in Broilers

**DOI:** 10.3390/ani12111471

**Published:** 2022-06-06

**Authors:** Seung-Gyu Moon, Damini Kothari, Woo-Do Lee, Jong-Il Kim, Kyung-Il Kim, Yong-Gi Kim, Gun-Whi Ga, Eun-Jip Kim, Soo-Ki Kim

**Affiliations:** 1 Department of Animal Science and Technology, Konkuk University, Seoul 05029, Korea; msg9807@gmail.com (S.-G.M.); damini.kth@gmail.com (D.K.); caw147@naver.com (W.-D.L.); rlawhddlf401@naver.com (J.-I.K.); kyungil3332@naver.com (K.-I.K.); ykkim@konkuk.ac.kr (Y.-G.K.); rkrjsgnl1@naver.com (G.-W.G.); 2 Division of Animal Husbandry, Yonam College, Cheonan 31005, Korea; yoosong11@naver.com

**Keywords:** *Paenibacillus konkukensis*, probiotic, feed additive, dietary, broiler

## Abstract

**Simple Summary:**

This study investigated the probiotic characteristics of *Paenibacillus konkukensis* and its dietary effects on growth performance, intestinal characteristics, intestinal microbiota, and meat quality in broilers. Through this study, *P. konkukensis* showed non-hemolytic activity, variable antibiotic susceptibility, and moderate acid and bile tolerance. Supplementary feeding of *P. konkukensis* as bacterial culture did not affect the growth performance parameters and meat quality, with positive effects on meat yield and intestinal microflora. Overall, dietary *P. konkukensis* may not have any adverse effects and might be considered as a probiotic feed additive in poultry.

**Abstract:**

This study evaluates the in vitro probiotic characteristics of *P. konkukensis* sp. nov. SK-3146, which was isolated from animal feed, and its dietary effects on growth performance, intestinal characteristics, intestinal microbiota, and meat quality in broilers. In vitro experiments revealed that *P. konkukensis* was non-hemolytic with variable antibiotic susceptibility, and acid as well as bile tolerance. To assess the effect of *P. konkukensis* on broilers, a total of four hundred eighty 1-day-old Ross 308 broiler chicks were allocated to 3 treatment groups with 4 replicates of 40 birds each; the negative control group was fed a basal diet without any feed additives (NC), the positive control group was fed a basal diet containing 0.01% enramycin (PC), and the experimental group was fed a basal diet containing *P. konkukensis* bacterial culture (PK) at 10^4^ CFU/g of the diet based on bacterial count. The experiment lasted for 35 days. Results indicated that there were no significant differences in any growth performance parameters among the dietary treatments (*p* > 0.05). In addition, the inclusion of *P. konkukensis* in the broilers’ diet did not affect meat cooking loss, color, and pH but increased the relative weight of breast meat (*p* < 0.05). The PK group showed heavier intestinal weight and shorter intestinal length than the NC group (*p* < 0.05). The ratio of the intestinal weight to length of jejunum was the highest in the PK group (*p* < 0.05). The PK group showed increased counts of *Streptococcus thermophilus* (*p* < 0.05) with no adverse effects of *P. konkukensis* on other intestinal microbiota in the jejunum. This study implies that *P. konkukensis* might have the potential to be applied as a probiotic feed additive in poultry.

## 1. Introduction

Since Moore’s study [1] first reported positive effects of antibiotics on growth performance in birds, non-therapeutic use of antibiotics has been studied for its beneficial effects on various livestock animals and permitted as in-feed antibiotics (IFA) to improve production efficiency of animals [2,3]. The use of IFA has played a crucial role in increasing the economic effectiveness of the animal industry [3]. However, the use of IFA in inappropriate dosages and during prolonged periods may increase the emergence of drug-resistant strains of pathogens [4,5,6]. Furthermore, the imbalance of intestinal microbial composition through destruction of beneficial bacteria by IFA inclusion has led to the frequent incidence of gastrointestinal diseases with sub-optimal animal performance and may have subsequently increased the consumption of therapeutic antibiotics. [7]. Thus, the inclusion of IFA for growth promotion, and not treating or preventing disease, has been banned worldwide [4], and the poultry industry has been aiming to use effective alternatives to antibiotics to reduce economic losses [4,8].

Probiotics are live microorganisms that, when administered in adequate amounts, confer a health benefit to the host. Through the currently reliable literature, well-studied and widely used probiotic microbial species are *Bifidobacterium* and *Lactobacillus* that are recommended for consumption for nutrition and health benefits [9]. In animals, probiotics are supplied to the host through feed to improve the host’s intestinal microbial environment by competitive exclusion and reduction of intestinal pH, which inhibits growth of pathogenic Gram-negative bacteria and increases the abundance of beneficial bacteria [10,11]. Additionally, several probiotic strains can produce various digestive enzymes that enhance the availability of feed nutrients to the host [10,12]. In poultry, probiotic feed additives have been reported to improve productivity, the epithelial membrane of the digestive tract, and meat quality [13]. A potentially probiotic strain fulfills functional considerations such as antimicrobial properties and useful metabolites or enzyme secretions, technological considerations such as viability in the gastrointestinal tract (GIT) and adhesion to the epithelium, and criteria for being generally recognized as safe (GRAS). Additionally, to prevent a risk through transfer of antibiotic resistance gene, a strain with antibiotic resistance cannot be approved as a probiotic strain [9,14,15].

*Paenibacillus* species are plant-related microorganisms that are spore-forming facultative anaerobes. These species have nitrogen-fixing activity, growth inhibitory activity against plant pathogens, and are mainly reported to promote plant growth [16]. In poultry, several previous studies [17,18,19,20,21] have reported that *Paenibacillus* spp. exhibit dietary functions as potential probiotics, including improvement of productivity and immunity. Recently, *P. xylanexedens* ysm1 in feed has been reported to be beneficial for broilers by improving the feed efficiency and morphological parameters of the digestive tract [17], and by improving the feed efficiency and body weight gain in broilers challenged with *Escherichia coli* K88 [18]. Another study indicated that *P. polymyxa* improved the intestinal health of broilers by enhancing intestinal epithelium, antioxidant status, immune responses, growth parameters, nutrient digestibility, and intestinal microflora [19,21,22]. In addition, *P. polymyxa* has also been reported to improve egg productivity, antioxidant status, and immune responses in Japanese quails [20].

In our previous studies [23,24], *P. konkukensis* sp. nov. SK-3146^T^ (=KACC 18876^T^ = LMG 29568^T^) was isolated from animal feed and completely sequenced. The strain has an abundance of genome sequences encoding various enzymes that improve digestion and the availability of nutrients in animal feed, such as arylsulfatase, protease, and xylanase. Furthermore, bacteriocin-encoding nucleotide sequences were also detected within the strain. The potential of the strain as a probiotic feed additive for livestock is high. There are no applied studies observing probiotic characteristics including safety of the strain and effects on animals. Therefore, the objective of this study was to investigate potential probiotic acceptability including hemolytic activity, antibiotic susceptibility, acid and bile tolerance, and adhesion characteristics of *P. konkukensis* and its dietary effect on growth performance, intestinal characteristics, intestinal microbiota, and meat quality in broilers.

## 2. Materials and Methods

### 2.1. In Vitro Experiments of Probiotic Acceptability

#### 2.1.1. Hemolytic Activity

The hemolytic activity of *P. konkukensis* was determined by using Reasoner’s 2A (R2A; Kisan Bio, Seoul, Korea) agar plate containing 5% (*v*/*v*) of horse blood. A single colony of *P. konkukensis* was streaked on the agar plate, and the activity was evaluated after overnight incubation at 37 °C. The hemolytic activity was classified according to whether red blood cells in the medium around the colonies were dissolved. The clear zone around colonies was interpreted as ß-hemolysis, the green or brown zone around colonies was interpreted as α-hemolysis, and the lack of zone around colonies was interpreted as γ-hemolysis.

#### 2.1.2. Antibiotic Susceptibility

The antibiotic susceptibility of *P. konkukensis* was determined according to the Kirby–Bauer disc diffusion method. The antibiotics used in this study were gentamycin (2 µg), vancomycin (30 µg), ampicillin (2 µg), tetracycline (30 µg), oxacillin (1 µg), kanamycin (64 µg), erythromycin (1 µg), chloramphenicol (30 µg), and clindamycin (2 µg) (Oxoid Ltd., Basingstoke, Hampshire, UK). The freshly prepared *P. konkukensis* bacterial culture incubated at 37 °C for 24 h was swabbed onto R2A agar plate using sterilized cotton swabs. Each disc of antibiotics was placed on the agar plate. After incubation at 37 °C for 48 h, a clear zone around the disc was observed.

#### 2.1.3. Acid and Bile Salt Tolerance

Acid tolerance was determined with R2A broth adjusted to 2.5, 3, and 3.5 pH using hydrochloric acid (12 M). For the adequate number of viable cells, *P. konkukensis* was inoculated in 60 mL of R2A broth, and the culture solutions were incubated at 37 °C for 48 h. Then, cell pellets were collected by centrifugation at 10,000× *g*, 4 °C for 5 min and washed with phosphate-buffered solution (PBS) (pH 7.2) twice. The pellets were resuspended in PBS to obtain an absorbance of 1.9 at 600 nm. Suspension of 500 µL was mixed with 5 mL of the pH-adjusted R2A broth and incubated for 1, 2, and 3 h. Cultures grown in R2A broth at pH 7 served as controls. Survival percentage was calculated by measuring the viable cell counts at 0, 1, 2, and 3 h of incubation using the drop plate method. Bile salt tolerance was determined with R2A broth supplemented with 0, 0.5, 1, and 2% (*w*/*v*) bile salt and incubated at 37 °C for 24 h. Survival percentage was evaluated by measuring the viable cell count at 0 h and 24 h of incubation using the drop plate method.

#### 2.1.4. Adhesion Assay

The hydrophobicity and auto-aggregation of *P. konkukensis* were determined as described by Das and Goyal [25] with some modifications using a UV-visible spectrophotometer (UV-1601, Shimadzu, Kyoto, Japan). According to the microbial adhesion to hydrocarbons (MATH) method, the hydrophobicity was measured using dodecane, toluene, and xylene as hydrocarbons. For the preparation of the test culture solution, *P. konkukensis* was incubated at 37 °C for 48 h. The cell pellets were washed twice with PBS and resuspended in PBS to an absorbance of 0.6 (A_0_) at 600 nm. Cell suspensions of 3 mL and 1 mL of hydrocarbon were vortexed for 60 s and incubated at 37 °C for 20 min for phase separation. The absorbance of the aqueous layer (A_t_) was measured at 600 nm, and the percent of bacterial adhesion to hydrocarbon was calculated by the following formula.

Hydrophobicity (%) = 1 − A_t_/A_0_ × 100

For the determination of auto-aggregation of *P. konkukensis*, the cell pellets were washed twice and resuspended in PBS to obtain an absorbance of 1.0 (A_0_) at 600 nm. The suspension of 3 mL was mixed by vortexing for 60 sec and incubated at 37 °C for 4 h. Afterward, the absorbance at 600 nm of the upper layer (A_t_) was measured. The percentage of auto-aggregation was calculated by the following formula.

Auto-aggregation (%) = 1 − A_t_/A_0_ × 100

### 2.2. Broiler Experiment

#### 2.2.1. Preparation of *P. konkukensis* Bacterial Culture

*P. konkukensis* stored at −80 °C with dimethyl sulfoxide as stock culture was inoculated on nutrient agar (Difco Laboratories, Detroit, MI, USA) and incubated at 37 °C for 24 h. Then, one colony was inoculated in 5 mL of nutrient broth and incubated at 37 °C for 20 h under shaking conditions (100 rpm), and then 1% of the culture was inoculated in 30 mL of nutrient broth and incubated in the same way as above. Finally, an adequate count in 600 mL of final culture solution was prepared by inoculating 1% of the 30 mL culture solution in nutrient broth and shaking incubation at 37 °C for 48 h. The *P. konkukensis* count in the final culture solution was 2.3 × 10^7^ CFU/mL.

#### 2.2.2. Experimental Animals, Diets, and Design

This experiment was designed with reference to our previous study [6]. A total of four hundred eighty 1-day-old male Ross 308 broilers were allocated to three treatment groups as follows: negative control group fed basal diet without additive (NC), positive control group fed basal diet containing 1 ppm of enramycin (PC), and the experimental group fed basal diet containing 0.1% (*v*/*w*) of *P. konkukensis* bacterial culture (PK), with 4 replicates (pens) per group of 40 birds each according to a completely randomly design. The dosage level of 0.1% of the bacterial culture was set in consideration of the inclusion level of the viable cells and the appropriate moisture content of the feed. All birds were vaccinated with Newcastle disease (ND) virus vaccine and housed in metal pens (1.8 m × 1.8 m) at a stocking density of 20 kg/m^2^. A corn-soybean meal basal diet was formulated with a nutritional level that met or exceeded the requirements of the 2017 Korean poultry feeding standard [26] (Table 1). A grower diet with 3100 kcal/kg of apparent metabolic energy (AMEn) and 20.5% crude protein (CP) and a finisher diet with 3150 kcal/kg of AMEn and 20% CP were used as basal diets from day 1 to 21 (grower phase) and day 21 to 35 (finisher phase), respectively. For preparing the experimental diet for the PK group, amounts of bacterial culture equivalent to 0.1% (*v*/*w*) of the diet (final bacterial count: 10^4^ CFU/g of the diet) were premixed with 10 kg of basal diet manually. Then, the premixed feed was mixed with the entire basal diet of the experimental diet for 10 min using a feed mixer (DKM-350SU, Daekwang Co., Ltd., Hwaseong, Gyeonggi-do, Korea). The antibiotic-containing diet for the PC group was prepared by supplementing 0.01% of ENRACIN-10 (WooGene B&G, Yeongdeungpo-gu, Seoul, Korea) to the basal diet and mixing similarly as above. After a 1-week adaptation period on a basal diet, birds were reallocated to equalize their initial body weights among the replicates within each treatment. Thereafter, the experimental diets were given for 4 weeks of the experimental period. During the entire experiment, feed in mash form and drinking water were provided ad libitum with 24 h of continuous artificial light. The temperature was maintained at 33 ± 2 °C for the adaptation period. Afterward, the temperature was gradually decreased by 2 °C every week until 26 °C was reached and maintained thereafter.

#### 2.2.3. Growth Performance

The body weight (BW) and feed intake (FI) were recorded weekly. All birds within a pen were weighed collectively to calculate the BW. The remaining feed was weighed, and the FI was calculated by subtracting its weight from the weight of the provided feed. For the calculation of BW, the weight of each pen was divided by the number of birds in each pen. Average daily gain (ADG) for each phase was calculated as the BW gain divided by the number of days. FI was calculated by subtracting the weight of the remaining feed from the weight of the feed offered on a weekly basis. The FI was divided by the number of days to obtain the average daily feed intake (ADFI), which was divided by the ADG to calculate the feed conversion ratio (FCR).

#### 2.2.4. Sample Collection and Measurements

At the end of the experiment, two birds per replicate were randomly selected and euthanized by CO_2_ asphyxiation to collect meat (breast and thigh) and intestine (duodenum, jejunum, ileum, and ceca). After removing mesentery and fat, the intestine and meat were sampled. The intestine was divided into four sections: pancreatic loop (duodenum); from the end of the pancreatic loop to Meckel’s diverticulum (jejunum); from the Meckel’s diverticulum to ileocecal junction (ileum); left and right ceca (cecum). Immediately, all samples were placed on ice and transported to the laboratory. After collecting the intestinal contents of each part of the intestine, the length and weight of each part of the intestine were measured and expressed as the ratio of length per 100 g of live body weight using an electronic balance (EL4002, Mettler Toledo, OH, USA). For obtaining microbial counts of intestinal microbiota accurately, the experiment was performed on the same day of collection. Total aerobic bacteria were counted on nutrient agar (Difco Laboratories, Detroit, MI, USA). Coliform and lactose-negative bacteria were counted on MacConkey agar (Difco Laboratories, Detroit, MI, USA). Lactic acid bacteria were counted on deMan Rogosa Sharpe (MRS, Burlington, MA, USA; Difco Laboratories, Detroit, MI, USA). *Streptococcus thermophilus* was counted on selective agar prepared as described by Dave and Shah [27]. The intestinal contents were diluted with sterilized distilled water in a ratio of 1:9 and serially diluted to reach a measurable count of bacteria. Therefore, an aliquot (10 μL) of suspensions was plated onto each medium with drop plate method in triplicate. Plates were incubated for 24 to 48 h at 37 °C. After incubation, colonies were counted and shown as log CFU/g. For meat quality, the weight of thigh meat from which bones were removed and breast meat was measured using an electronic balance and expressed as the weight per 100 g of live weight. After the sample was shaped into a constant shape, it was placed in a polyethylene bag and heated in a 75 °C water bath (C-WBE, Chang Shin co., Hwaseong, Korea) for 30 min. Then, it was allowed to cool for 10 min at room temperature, and the cooking loss (%) was calculated using the following formula.

Cooking loss (%) = (Sample weight before cooking − Sample weight after cooking)/(Sample weight before cooking) × 100

The meat color was measured using a colorimeter (Chromameter, CR210, Minolta, Japan) on the surface of the sample. L* (lightness), a* (redness), and b* (yellowness) were obtained. The pH was calculated as a value measured with a pH meter (HI-98163, Hanna Instruments, Nusfalau, Romania) at a depth of 1 cm of the breast and thigh meat and was measured three times per sample.

### 2.3. Statistical Analysis

IBM SPSS statistics 25 (SPSS Inc., Chicago, IL, USA) was used for in vitro data analyses. Paired t-test was used to study significant difference between means, with significance level at *p* < 0.05. The PROC MIXED procedure of Statistical Analysis System 9.4 (SAS, Cary, NC, USA) was used considering each replicate pen as the experimental unit for growth performance. The data on intestine, intestinal microbiota, and meat were analyzed while considering each collected bird as the experimental unit. Differential test among means was conducted with one-way ANOVA using Tukey’s test. *p* < 0.05 was considered statistically significant. The results were presented as means ± standard error of means (SEM).

## 3. Results

### 3.1. Hemolytic Activity and Antibiotic Susceptibility

*P. konkukensis* showed γ-hemolytic activity (results not shown). The antibiotic susceptibility of *P. konkukensis* is shown in Table 2. Except for oxacillin, *P. konkukensis* was susceptible to all tested antibiotics.

### 3.2. Acid and Bile Salt Tolerance and Adhesion Properties

**Figure 1:**
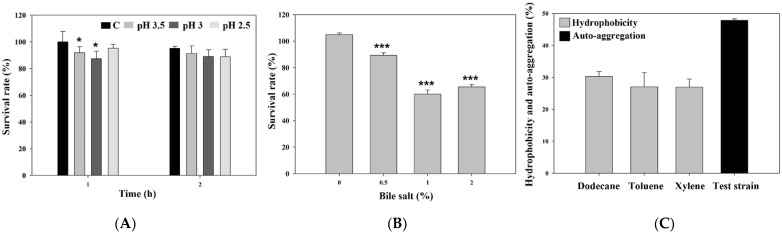
Acid tolerance (**A**), bile salt tolerance (**B**), and hydrophobicity and auto aggregation (**C**) of *P. konkukensis*. * *p* < 0.05 and *** *p* < 0.001.

The survival rate after 1 h of incubation in the medium adjusted to pH 3.5 and 3 was significantly lower than that in the control (*p* < 0.05; Figure 1A). However, after 2 h of incubation, any treatments of pH-adjusted medium did not show a significantly different survival rate from the control. The survival rate after 24 h of incubation in the medium containing bile salt was significantly lower in the 0.5, 1, and 2% containing medium than in the 0% containing medium (*p* < 0.001; Figure 1B).

Hydrophobicity of *P. konkukensis* against dodecane, toluene, and xylene was 30.29, 27.08, and 26.92%, respectively. However, a moderate auto-aggregation value of 47.88% was shown in this study [Figure 1C].

### 3.3. Growth Performance

There were no significant differences in any of the growth performance parameters, including BW, BW gain, ADG, ADFI, and FCR, among the treatment groups (*p* > 0.05; Table 3).

### 3.4. Intestinal Weight and Length

The weight of the duodenum, jejunum, and ileum was significantly higher in the PK group as compared to the PC group (*p* < 0.05; Table 4). The length of the duodenum, jejunum, and ileum was significantly shorter in the PC or PK group compared to the NC group (*p* < 0.05). For the ratio of weight to length, the PK group showed a significantly higher ratio than that of the PC group in the duodenum and ileum (*p* < 0.05). In the jejunum, the ratio of the PK group was significantly higher than that of the PC or NC group (*p* < 0.05). However, no significant differences were found among the treatment groups in the cecum for all parameters (*p* > 0.05).

### 3.5. Intestinal Microbiota

In the jejunum, the counts of coliform and lactose-negative enterobacteria were significantly higher in the PC group as compared to the NC group (*p* < 0.05; Table 5). However, the counts of lactobacilli and total aerobes in the PC group were decreased as compared to the NC and PK groups (*p* < 0.01). The counts of *S. thermophilus* were significantly highest in the PK group among all the treatment groups. However, no significant differences were found in the ileum for all types of microbes studied (*p* > 0.05). In addition, the counts of LAB in the cecum were significantly lower in the PC group than in the NC group (*p* < 0.05).

### 3.6. Meat Quality

The a* value of thigh meat was significantly lowest in the PC group among the treatment groups (*p* < 0.01; Table 6). The relative weight of breast meat to BW was significantly higher in the PK group than in the other treatment groups (*p* < 0.05). Except for the above results, there were no significant differences in cooking loss and pH among the treatment groups (*p* > 0.05).

## 4. Discussion

Determination of hemolytic activity is one of the considerations for confirming the safety of potential probiotic strains according to the guidelines from FAO and WHO [28]. In this study, *P. konkukensis* showed γ-hemolytic activity, and thus was considered safe to use for probiotics. Antibiotic susceptibility is another important trait for selecting probiotic strains. Probiotic strains should not possess resistance to clinical antibiotics and have transmissible antibiotic resistance that can lead to the development of new antibiotic-resistant pathogens [29]. Resistance to multiple antibiotics in many species of *Paenibacillus* has been reported by several studies [30,31]. However, *P. konkukensis* showed high sensitivity to all tested antibiotics except for oxacillin.

One of the most important characteristics of a probiotic strain is its potential to tolerate and withstand the acidic stomach conditions to reach the intestine. The pH in chicken gastrointestinal tract ranged from 2.5 to 4.7, and digestion could take from 1 up to 3 h depending on the size of the feed [32]. Although the viability of *P. konkukensis* after 1 h of incubation was significantly decreased in specific pH-adjusted conditions (3 and 3.5), the viability after 2 h of incubation was maintained from 88.9 to 95.2% in all pH-adjusted conditions. The presence of bile salts in the intestines of animals is another important factor that a potential probiotic strain must tolerate in order to reach the intestine [33]. The total bile salt concentration in the chicken gastrointestinal tract ranges from 0.008 to 0.175% [34]. However, many studies considered the level of bile salt tolerance for potential probiotic strains at the average level of 0.3% bile salt [35,36]. In our study, the survival rate of *P. konkukensis* was above 80% in 0.5% bile salt after 24 h of incubation. The good acid and bile salt resistance of *P. konkukensis* might be explained by characteristics of Gram-positive bacteria including acid tolerance systems that can help them to overcome the challenge posed by different acidic environments [37] and the presence of bile salt hydrolase activity, which can hydrolyze bile and minimize its bactericidal effect [38]. However, there are few studies reporting acid and bile salt tolerance and their mechanisms, especially in *Paenibacillus* species, and thus, further confirmation of the tolerances is required.

Adhesion and aggregation are properties that indicate the degree of colonization on the intestinal epithelium and mucosal surface. These probiotic properties are required to compete with enteropathogenic bacteria by forming biofilm or modulating the host’s immune response [28]. Auto-aggregation and hydrophobicity have a significant positive relationship, and both properties are closely related to the ability to adhere to the cell surface. Although *P. konkukensis* showed a high auto-aggregation value, the hydrophobicity was lower than 40%, which is the minimum value that can be considered for a probiotic strain [25]. These results indicate that *P. konkukensis* may have little capacity to create an antibacterial environment in the intestine while adherent to intestinal epithelial cells.

Several previous studies [17,18,19,21,39] have indicated that *Paenibacillus* species have improved BW gain and feed efficiency in broilers as probiotic availability for feed additives has also been reported. Wang et al. [20] reported that the intestinal digestive environment was improved, and feed efficiency was increased slightly in broilers fed with *P. polymyxa* 10. Another study [22] reported that fermentation with *P. polymyxa* reduced the crude fiber content in palm kernel cake and its fermented form improved the feed efficiency in broilers as feed ingredient when compared with the non-fermented form. In addition, probiotic feeding of *P. xylanexedens*, which secretes enzymes such as D-cellulose, D-fructose, D-galactose, α-D-glucose, lactose, and maltose as actively as *Bacillus* species, improved the BW gain and FCR in broilers under normal feeding environment [17] or challenged conditions [18]. Similarly, *P. konkukensis*, isolated and analyzed for complete genome sequence in our previous studies, has DNA sequences encoding various types of enzymes such as β-glucosidase, cellulase, xylanase, and protease, which can degrade various carbohydrates and anti-nutritional factors [23,24]. However, in this study, *P. konkukensis* had no measured effects on the growth parameters. The strain may not have secreted enough extracellular enzymes to improve bioavailability of feed nutrients, which would appear as a change in growth parameters in broilers during the experimental period. Another reason might be ascribed to the fact that the viable count of *P. konkukensis* was too low to induce any significant effect on growth performance even after 7 days of age, when the intestinal microflora is not easily affected by probiotic additives [40]. Further research is required for detailed observation of changes in nutrient digestibility in broilers fed with the strain with a high viable cell count from 1 day of age.

The size and structure of the intestinal tract are useful indicators to predict the effect of feed components on the function and development of organs in birds. The longer intestine increases the energy required to maintain itself and ultimately reduces the energy input to the production activities. Furthermore, the longer retention time of digesta required for the digestive process in the intestine further increases the length of the intestinal tract [41]. Therefore, the feed efficiency and nutrient digestibility are increased concomitantly with reduced intestinal length and weight [42]. However, while the PK group showed decreased intestinal length versus the PC group, no differences in the growth performance and feed efficiency were identified. Furthermore, the increased intestinal weight compared to the length in the PK group was an inconsistent result. Thus, further studies are imperative to observe the developmental status of intestinal epithelium and thickness of the intestine resulting in changes in intestinal length and weight.

Probiotic feed additives in poultry have beneficial effects including stabilization of the gut microflora and immune function. An intestinal microenvironment where beneficial microorganisms are increased and the colonization of pathogenic bacteria is inhibited can be formed with the help of probiotics [3,10]. Many studies indicated that dietary supplementation with probiotics improved intestinal microflora. For instance, dietary supplementation with *B. subtilis* decreased *E. coli* populations in the ileum and cecum of broilers [43]. The inclusion of *L. johnsonii* in feed promoted intestinal microflora by increasing the population of Bacteroidetes and *Lactobacillus* spp. and reducing the population of Enterobacteriaceae and Firmicutes in broilers [44]. In studies including *Paenibacillus* strains, *P. polymyxa* with antibacterial activity against pathogenic bacterial strains reduced the *E. coli* and *Enterococcus* spp. in cecal microbiota of Japanese quail [20] and decreased ileal *Enterobacteriaceae* counts in broilers when the strain was fed in fermented form with palm kernel cake [24]. Another study reported that *P. xylanexedens* ysm 1 with antibacterial activity against *E. coli* reduced the cecal *E. coli* in broilers challenged with *E. coli* K88 [18]. Contrary to the adverse effects of antibiotics on the lactobacilli and coliform and lactose-negative enterobacteria, administration of *P. konkukensis* increased the population of ST and did not have any adverse effect on the populations of other types of intestinal microorganism in this study. Probiotic strains that secrete antibacterial substances, inhibit bacterial colonization on host epithelial cells, and compete for nutrients with the undesired bacteria stabilize the host’s intestinal microbiota in favor of beneficial bacteria [3,10]. However, within this study, *P. konkukensis* did not appear to create a harmful intestinal environment for the undesired bacteria. The reason may be speculated to be the absence of antimicrobial activity and insufficient adhesion activity of *P. konkukensis* identified in this study. From post hatch to 3 days of age, when the intestinal microflora is highly variable and being established, providing initial inoculum immediately can effectively establish the inoculum in the poultry gut microbiota. The delay due to starting the experimental period at 7 days of age may have induced the unaffected intestinal microflora in this study.

Among meat quality traits, meat color is an important meat quality trait that consumers use as an indicator for selection of overall fresh and wholesome meat products [45]. The high water-holding capacity (WHC), such as drip loss and cooking loss, means low loss of nutrients during exudation of water from meat [46]. Meat pH has a positive relationship with WHC and can negatively affect the meat color if it declines from an initial pH value to an ultimate pH value at postmortem abnormally [47]. In the study by Alshelmani et al. [22], palm kernel cake fermented by *P. polymyxa* did not affect the meat color, drip loss, cooking loss, and tenderness in broilers. Similarly, dietary supplementation with *P. konkukensis* in this study did not change the meat quality parameters, including cooking loss, color, and pH. The pH is closely related to other quality traits as mentioned above. In addition, different types of probiotic microorganisms have different effects on the meat pH, and the mechanism remains unclear [47]. We speculate that *P. konkukensis* might not affect the pH, and therefore, not influence other quality traits including cooking loss and color. In a few previous studies, probiotics were found to increase meat yield in broilers. Hossain et al. [48] reported that multi-strain probiotics in fermented form with *Alisma canaliculatum* increased relative breast meat weight to body weight. Hussein et al. [49] found that yeast and multi-strain probiotics in combination increased dressing ratio to body weight. Novak et al. [50] reported that *B. cereus* var. toyoi was effective in increasing meat yields in broilers. In the present study, the increased breast meat yield could be reflected in the improved dressing ratio to body weight. However, the reason for the low redness (a*) of thigh meat in the antibiotic treatment group is not clear.

## 5. Conclusions

*P. konkukensis* showed suitable in vitro probiotic characteristics including being non-hemolytic and having acceptable antibiotic susceptibility and moderate acid as well as bile tolerance. In broilers, dietary supplementation with *P. konkukensis* (10^4^ CFU/g of the diet) had positive effects on intestinal microbiota and breast meat yield. This study suggests that *P. konkukensis* may not have any adverse effects and might be considered as a probiotic feed additive for poultry. However, the small number of replicates, low inclusion level of bacterial cells of the strain, and absence of treatments using various dosages were the main drawbacks of this study. Therefore, further large-scale and dosage response experiments are needed to affirm the acceptability of *P. konkukensis* as a probiotic strain for poultry.

## Figures and Tables

**Table 1:** Ingredients and nutrient composition of the basal diet.

Item	Grower Phase (Day 1 to 21)	Finisher Phase (Day 21 to 35)
Ingredient composition, %		
Corn	57.2	52.53
Soybean meal (crude protein, 45%)	27.09	26.48
Rice bran	-	3
Corn gluten meal	3.38	3
Corn dried distillers’ grains with solubles (DDGS)	3	3
Tallow	3	5
Rapeseed meal	2	2
Wheat bran	-	1.25
Limestone	1.69	1.79
Monocalcium phosphate (MCP)	1.16	0.91
L-lysine HCl 78%	0.38	0.19
DL-methionine 98%	0.36	0.17
Salt	0.25	0.25
Choline-Cl 50%	0.09	0.04
Mineral premix ^(1)^	0.15	0.15
Vitamin premix ^(2)^	0.12	0.11
Phytase optis	0.05	0.05
NaHCO_3_	0.08	0.08
Calculated analysis		
Crude protein, %	20.5	20.0
Crude fat, %	5.84	8.23
Crude fiber, %	2.8	2.92
Ash, %	4.74	4.97
Ca, %	0.80	0.90
Available P, %	0.62	0.61
Met+Cys, %	1.03	0.83
AMEn (kcal/kg)	3100	3150

^(1)^ Mineral premix provided the following per kg of diet: Fe, 80 mg; Zn, 50 mg; Mn, 60 mg; Co, 0.3 mg; Cu, 10 mg; Se, 0.2 mg. ^(2)^ Vitamin premix provided the following per kg of diet: vitamin A, 80,000 IU; vitamin D_3_, 1600 IU; vitamin E, 20 IU; vitamin K_3_, 8 mg; vitamin B_1_, 8 mg; vitamin B_2_, 24 mg; vitamin B_6_, 12 mg; vitamin B_12_, 0.040 mg; pantothenic acid, 40 mg; folic acid, 4 mg; nicotinic acid, 120 mg.

**Table 2:** Antibiotic susceptibility of *P. konkukensis*.

Item	Diameter (mm)	Sensitivity
Gentamycin	13	S
Vancomycin	34	S
Ampicillin	14	S
Chloramphenicol	40	S
Clindamycin	40	S
Erythromycin	40	S
Oxacillin	ND	R
Tetracycline	20	S
Kanamycin	15	S

Inhibition zone diameter <8 mm, resistant (R); inhibition zone diameter between 8–10 mm, moderate (M); and inhibition zone diameter >10 mm, susceptibility (S).

**Table 3:** Dietary supplemental effect of *P. konkukensis* on growth performance in broilers.

Item	Treatment			*p*-Value
	NC	PC	PK	
BW (g)				
Day 7 (initial weight)	134.46 ± 0.04	134.41 ± 0.06	134.49 ± 0.03	0.39
Day 21	664.25 ± 19.61	704.44 ± 26.99	685.89 ± 18.37	0.46
Day 35	1536.63 ± 56.16	1618.06 ± 54.29	1573.13 ± 60.85	0.62
BW gain (g/day/bird)				
Grower phase	529.79 ± 19.59	570.03 ± 26.95	551.40 ± 18.37	0.46
Finisher phase	872.38 ± 39.18	913.63 ± 31.12	887.24 ± 44.22	0.75
Total phase	1402.16 ± 56.114	1483.66 ± 54.27	1438.64 ± 60.85	0.62
ADG (g/day/bird)				
Grower phase	37.84 ± 1.40	40.72 ± 1.93	39.39 ± 1.31	0.46
Finisher phase	62.31 ± 2.80	65.26 ± 2.22	63.37 ± 3.16	0.75
Total phase	50.08 ± 2.dd00	52.99 ± 1.94	51.38 ± 2.17	0.62
ADFI (g/day/bird)				
Grower phase	63.13 ± 1.10	62.75 ± 2.64	64.58 ± 3.07	0.85
Finisher phase	125.28 ± 2.97	129.94 ± 3.10	128.35 ± 4.09	0.63
Total phase	94.20 ± 1.69	96.34 ± 2.52	96.47 ± 3.57	0.81
FCR				
Grower phase	1.67 ± 0.04	1.54 ± 0.03	1.65 ± 0.12	0.45
Finisher phase	2.02 ± 0.05	1.99 ± 0.02	2.04 ± 0.13	0.91
Total phase	1.89 ± 0.04	1.82 ± 0.02	1.89 ± 0.12	0.77

Error of mean; NC: negative control, basal diet; PC: basal diet containing 1 ppm of enramycin; PK: basal diet + 0.1% *P. konkukensis* bacterial culture. Grower phase: day 7 to 21; Finisher phase: day 21 to 35: Total phase: day 7 to 35. BW: body weight; ADG: average daily gain; ADFI: average daily feed intake; FCR: feed conversion ratio.

**Table 4:** Dietary supplemental effect of *P. konkukensis* on intestinal weight and length in broilers.

Item	Treatment			*p*-Value
	NC	PC	PK	
Relative weight, g/100 g BW				
Duodenum	0.58 ^ab^ ± 0.04	0.45 ^b^ ± 0.03	0.65 ^a^ ± 0.04	<0.01
Jejunum	0.95 ^ab^ ± 0.07	0.71 ^b^ ± 0.03	1.04 ^a^ ± 0.09	0.01
Ileum	0.92 ^ab^ ± 0.08	0.70 ^b^ ± 0.04	0.94 ^a^ ± 0.08	0.03
Cecum	0.19 ± 0.04	0.17 ± 0.01	0.16 ± 0.02	0.64
Relative length, cm/100 g BW				
Duodenum	2.10 ^a^ ± 0.10	1.78 ^b^ ± 0.05	1.83 ^b^ ± 0.06	0.01
Jejunum	5.03 ^a^ ± 0.25	4.13 ^b^ ± 0.18	4.16 ^b^ ± 0.20	0.01
Ileum	5.57 ^a^ ± 0.24	4.55 ^b^ ± 0.21	4.55 ^b^ ± 0.24	<0.01
Cecum	1.13 ± 0.04	1.37 ± 0.26	1.07 ± 0.06	0.36
Weight/length, g/cm				
Duodenum	0.28 ^ab^ ± 0.03	0.25 ^b^ ± 0.02	0.35 ^a^ ± 0.02	0.02
Jejunum	0.19 ^b^ ± 0.02	0.17 ^b^ ± 0.01	0.25 ^a^ ± 0.02	<0.01
Ileum	0.17 ^ab^ ± 0.02	0.16 ^b^ ± 0.01	0.21 ^a^ ± 0.02	0.05
Cecum	0.17 ± 0.03	0.14 ± 0.02	0.15 ± 0.01	0.61

^a,b^ Means within the same row with different letters differ significantly at *p* ≤ 0.05. SEM: standard error of mean; NC: negative control, basal diet; PC: basal diet containing 1 ppm of enramycin; PK: basal diet + 0.1% *P. konkukensis* bacterial culture.

**Table 5:** Dietary supplemental effect of *P. konkukensis* on intestinal microbiota in broilers.

Type	Treatment (Log CFU/g)			*p*-Value
	NC	PC	PK	
Jejunum				
Coliform and lactose-negative enterobacteria	6.52 ^b^ ± 0.27	7.36 ^a^ ± 0.17	7.16 ^ab^ ± 0.19	0.04
Lactobacilli	8.41 ^a^ ± 0.13	7.60 ^b^ ± 0.23	8.86 ^a^ ± 0.12	<0.01
Total aerobes	8.05 ^a^ ± 0.15	6.87 ^b^ ± 0.23	7.77 ^a^ ± 0.18	<0.01
ST	7.03 ^b^ ± 0.31	6.81 ^b^ ± 0.16	8.41 ^a^ ± 0.18	<0.01
Ileum				
Coliform and lactose-negative enterobacteria	6.70 ± 0.34	7.58 ± 0.41	7.22 ± 0.20	0.20
Lactobacilli	8.95 ± 0.13	8.65 ± 0.20	8.87 ± 0.12	0.40
Total aerobes	7.51 ± 0.31	8.32 ± 0.23	8.38 ± 0.20	0.05
ST	7.88 ± 0.34	8.26 ± 0.11	8.30 ± 0.15	0.37
Cecum				
Coliform and lactose-negative enterobacteria	7.60 ± 0.21	7.56 ± 0.38	6.94 ± 0.16	0.13
Lactobacilli	9.27 ^a^ ± 0.10	8.50 ^b^ ± 0.31	9.20 ^ab^ ± 0.16	0.02
Total aerobes	7.80 ± 0.23	8.16 ± 0.35	7.65 ± 0.19	0.35
ST	9.00 ± 0.24	8.57 ± 0.34	9.08 ± 0.23	0.33

^a,b^ Means within the same row with different letters differ significantly at *p* ≤ 0.05; SEM: standard error of mean; NC: negative control, basal diet; PC: basal diet containing 1 ppm of enramycin; PK: basal diet + 0.1% *P. konkukensis* bacterial culture.

**Table 6:** Dietary supplemental effect of *P. konkukensis* on meat quality in broilers.

Type		Treatment (Log CFU/g)			*p*-Value
		NC	PC	PK	
Cooking loss, %					
Breast		68.00 ± 1.48	69.31 ± 1.10	70.11 ± 1.18	0.50
Thigh		68.57 ± 1.04	68.83 ± 1.01	70.68 ± 1.59	0.44
Meat color					
Breast	L*	57.75 ± 1.06	56.26 ± 1.77	59.44 ± 1.39	0.31
	a*	2.58 ± 0.38	2.48 ± 0.40	2.28 ± 0.54	0.89
	b*	12.17 ± 0.51	13.31 ± 0.53	13.59 ± 0.68	0.21
Thigh	L*	55.14 ± 0.61	56.52 ± 0.64	56.72 ± 0.34	0.11
	a*	14.46 ^a^ ± 0.28	12.07 ^b^ ± 0.45	13.60 ^a^ ± 0.41	<0.01
	b*	6.30 ± 0.43	7.43 ± 0.33	6.19 ± 0.35	0.06
pH					
Breast		5.59 ± 0.04	5.66 ± 0.03	5.54 ± 0.05	0.16
Thigh		6.10 ± 0.10	6.26 ± 0.04	6.15 ± 0.04	0.20
Meat weight, g/100 g BW					
Breast		6.43 ^b^ ± 0.13	6.51 ^b^ ± 0.09	7.37 ^a^ ± 0.27	<0.01
Thigh		6.43 ± 0.13	6.51 ± 0.09	6.60 ± 0.15	0.62

^a,b^ Means within the same row with different letters differ significantly at *p* ≤ 0.05; SEM: standard error of mean; NC: negative control, basal diet; PC: basal diet containing 1 ppm of enramycin; PK: basal diet + 0.1% *P. konkukensis* bacterial culture.

## Data Availability

The data presented in this study are available in this paper.
